# Simultaneous extraction and detection of peptides, steroids, and proteins in small tissue samples

**DOI:** 10.3389/fendo.2023.1266985

**Published:** 2023-10-09

**Authors:** Chunyu Lu, Di Peng, W. C. K. Udeesha Erandani, Kimberly Mitchell, Christopher J. Martyniuk, Vance L. Trudeau

**Affiliations:** ^1^ Department of Biology, University of Ottawa, Ottawa, ON, Canada; ^2^ Department of Physiological Sciences, University of Florida, Gainesville, FL, United States

**Keywords:** protein measurement, peptide quantification, steroid detection, LCMS (liquid chromatography-mass spectrometry), solid phase extraction

## Abstract

The detection and quantification of hormones are important to assess the reproductive and stress status of experimental models and for the diagnosis of diseases in human and veterinary clinics. Traditionally, steroid, peptide, and protein hormones are analyzed in individual experiments using different extraction methodologies. With the new advancement on HPLC sorbents, the simultaneous measurement of hormones from different categories becomes possible. In this study, we present a novel sample processing strategy for the simultaneous extraction and detection of peptides, steroids, and proteins using high-resolution liquid chromatography tandem mass spectrometry. We demonstrate the sensitivity of our method for small tissues by acquiring data from brain, pituitary gland, and gonads of single zebrafish samples. This approach promises to shed light on the hormonal pathways and their interrelationships, providing knowledge on the integration of hormone systems.

## 1 Introduction

Measuring peptides, steroids, and proteins from tissue extracts is essential for studying normal physiological processes and diagnosing various pathological states with underlying endocrine etiologies. Liquid chromatography-tandem mass spectrometry (LC-MS/MS) is the technique of choice for qualitative and quantitative hormone measurements because of critical advances in analytical chemistry. However, the diverse chemical structures of peptides, steroids, and proteins have traditionally required separate LC-MS/MS analyses of different biological samples, which limits project scope and the ability to simultaneously collect integrated data (1–3). For peptide and steroid methodologies, proteins are typically precipitated out of extracts and discarded, while for proteomics, the protein analytes are precipitated and preserved, and the supernatants are discarded (4–6). These incompatible approaches have limited comprehensive evaluation and pose significant challenges for data interpretation. Simultaneous measurement of these diverse analytes would represent a major advancement in endocrine research. Direct comparison of variations and interrelationships for numerous hormones in a single sample could reveal critical physiological mechanisms or underlying causes of disease.

**Figure 1:**
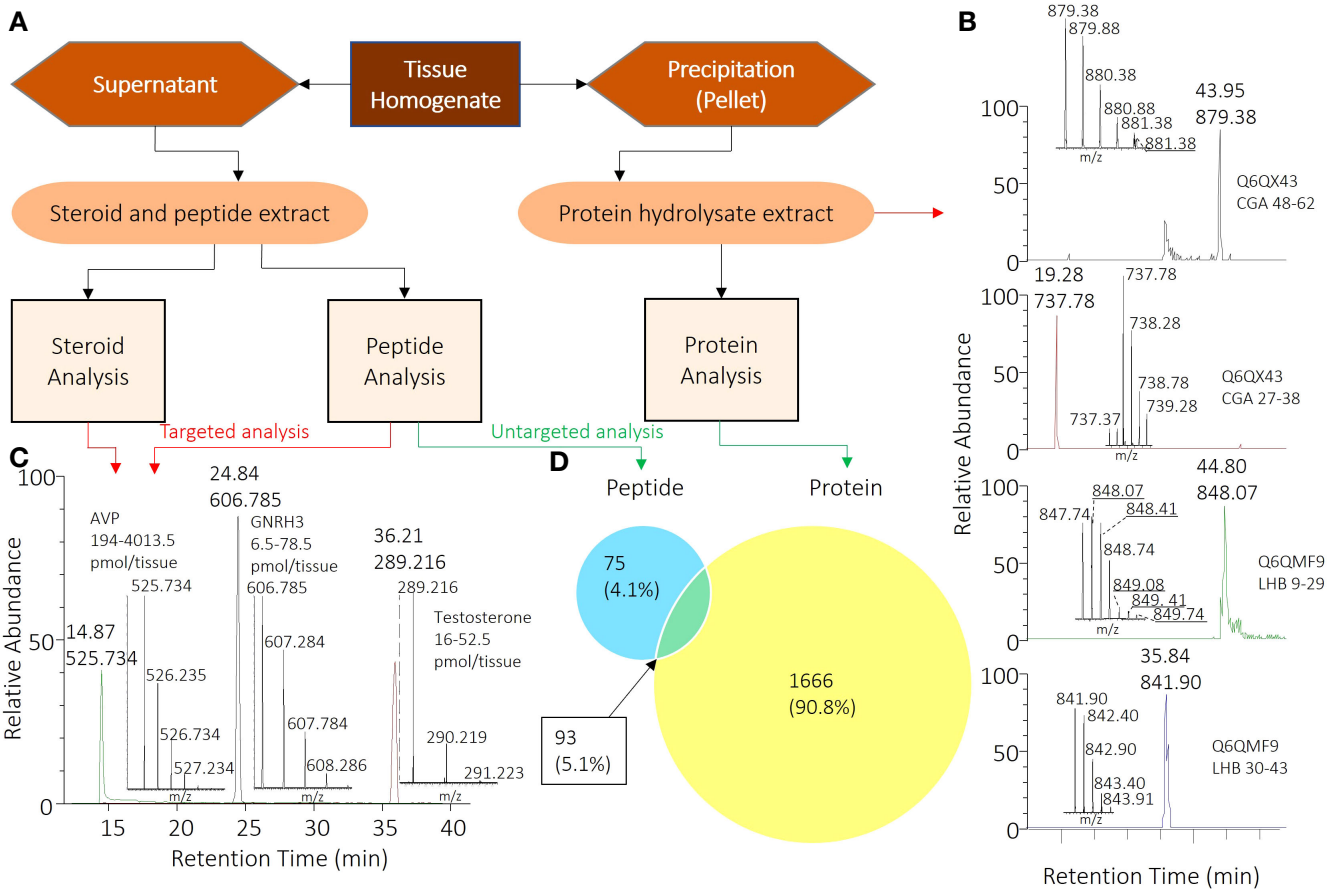
Comprehensive Workflow for Analyzing Peptide, Steroid, and Protein Components in Pituitary Samples. **(A)** Overall sample processing workflow from tissue homogenate to specific samples for corresponding analyses. **(B)** Chromatogram and mass spectrometry results for CGA and LHB) peptides selected for targeted proteome analysis. **(C)** Chromatogram and mass spectrometry results for selected peptide and steroid analyses, with AVP, GNRH3, and testosterone serving as model compounds among all analytes. **(D)** 93 pituitary proteins (5.1%) that were identified with both peptidomics and trypsin-digested proteomics. Also shown are the numbers of other peptides (75; 4.1%) and proteins (1666; 90.8%) identified.

The emergence of high-quality high-performance liquid chromatography (HPLC) sorbents has made it possible to address the long-standing issue of co-extraction and analysis. Our approach employs a unique extraction strategy, which uses a reverse phase sorbent initially intended for ultra-high-performance liquid chromatography (UHPLC). The homogenization solution consists of a high percentage of methanol and acetic acid to precipitate proteins from tissue homogenates, while keeping peptide and steroid analytes in solution. This enables the digestion of the protein pellet for bottom-up proteomics. The supernatant containing peptides and steroids can be safely stored for subsequent experiments (**Figure 1**).

To test the method, we employed the zebrafish (*Danio rerio*), an emerging small model organism for the study of reproductive biology and endocrine diseases (7–9). We have been able to compare the levels of well-known hormones with those of the newest reproductive hormone secretoneurin, which we have reported on recently (10). We show that it is possible to perform both proteomics and peptidomics on the same small tissue sample, revealing the intricate relationships between precursor and product. In addition, we have succeeded in extracting peptides, steroids, and proteins from a single and very small tissue, the pituitary gland of the zebrafish. The processing procedures were optimized for small sample volume using a 96-well plate platform.

## 2 Materials and equipment

### 2.1 Reagents and materials

• 10 µm sorbent (Dr. Maisch HPLC GmbH, cat. no. r13.aq.0010)
• 3 µm sorbent (Dr. Maisch HPLC GmbH, cat. no. r13.aq.0003)
• Filtered 96-well plate (OROCHEM, cat. no. OF1100)
• Estrone (Cayman Chemical, cat. no. 10006485)
• Estradiol (Cayman Chemical, cat. no. 10006315)
• Estriol (Cayman Chemical, cat. no. 10006484)
• Testosterone (Cayman Chemical, cat. no. 15645)
• 11-Ketotestosterone (Cayman Chemical, cat. no. 9002564)
• Fmoc-Ala-OH (Cayman Chemical, cat. no. 41004)
• Fmoc-Arg(Pbf)-OH (P3 BioSystems, cat. no. 41002)
• Fmoc-Asn(Trt)-OH (P3 BioSystems, cat. no. 41007)
• Fmoc-Asp(OtBu)-OH (P3 BioSystems, cat. no. 41019)
• Fmoc-Cys(Trt)-OH (P3 BioSystems, cat. no. 41008)
• Fmoc-Cys(Acm)-OH (P3 BioSystems, cat. no. 41063)
• Fmoc-Glu(OtBu)-OH (P3 BioSystems, cat. no. 41005)
• Fmoc-Gln(Trt)-OH (P3 BioSystems, cat. no. 41011)
• Fmoc-Gly-OH (P3 BioSystems, cat. no. 41010)
• Fmoc-His(Trt)-OH (P3 BioSystems, cat. no. 41017)
• Fmoc-Ile-OH (P3 BioSystems, cat. no. 41018)
• Fmoc-Leu-OH (P3 BioSystems, cat. no. 41003)
• Fmoc-Lys(Boc)-OH (P3 BioSystems, cat. no. 41001)
• Fmoc-Met-OH (P3 BioSystems, cat. no. 41020)
• Fmoc-Phe-OH (P3 BioSystems, cat. no. 41013)
• Fmoc-Pro-OH (P3 BioSystems, cat. no. 41009)
• Fmoc-Ser(tBu)-OH (P3 BioSystems, cat. no. 41006)
• Fmoc-Thr(tBu)-OH (P3 BioSystems, cat. no. 41016)
• Fmoc-Trp(Boc)-OH (P3 BioSystems, cat. no. 41012)
• Fmoc-Tyr(tBu)-OH (P3 BioSystems, cat. no. 41014)
• Fmoc-Val-OH (P3 BioSystems, cat. no. 41015)
• Boc-Pyr-OH (P3 BioSystems, cat. no. 47222)
• (2- (1 H -benzotriazol-1-yl)-1,1,3,3-tetramethyluronium hexafluorophosphate (HBTU) (P3 BioSystems, cat. no. 31001)
• Tentagel resin (Rapp Polymere GmBH, cat. no. S3023)
• N-methylmorpholine (NMM) (Millipore sigma, cat. no. M56557)
• Trifluoroacetic acid (TFA) (Millipore sigma, cat. no. T6508)
• Formic acid (FA) (Millipore sigma, cat. no. 695076)
• Triisopropylsilane (Millipore sigma, cat. no. 233781)
• Piperidine (Millipore sigma, cat. no. 104094)
• Iodine (Millipore sigma, cat. no. 207772)
• Acetonitrile (ACN) (Fisher Scientific, cat. no. A9984)
• Methanol (MeOH) (Fisher Scientific, cat. no. BP1105-4)
• Dimethylformamide (DMF) (Fisher Scientific, cat. no. BPD1194)
• N-methyl-2-pyrrolidone (NMP) (Millipore sigma, cat. no. 8060721000)
• Urea (Millipore sigma, cat. no. 9510-OP)
• Ammonium bicarbonate (Millipore sigma, cat. no. A6141)
• Dithiothreitol (DTT) (Millipore sigma, cat. no. D9163)
• Iodoacetamide (Millipore sigma, cat. no. A3221-10VL)
• Ammonium fluoride (NH4F) (Millipore sigma, cat. no. 338869-25G)
• Trypsin (Worthington-biochemical, cat. no. LS02120)
• Milli-Q water

### 2.2 Recipes for solutions used in the protocol

#### 2.2.1 Homogenization solution

▪ 90% MeOH
▪ 9% water
▪ 1% acetic acid

#### 2.2.2 Activation solution

▪ 0.2% FA
▪ 50% ACN
▪ 50% water

#### 2.2.3 Equilibrium solution

▪ 0.2% FA
▪ 5% ACN
▪ 95% water

#### 2.2.4 Elution solution

▪ 0.2% FA
▪ 75% ACN
▪ 25% water

#### 2.2.5 Mobile phase A

▪ 0.1% FA in water

#### 2.2.6 Mobile phase B

▪ 0.1% FA in ACN

#### 2.2.7 Mobile phase A1

▪ 0.2% FA in water

#### 2.2.8 Mobile phase B1

▪ 0.2% FA
▪ 80% ACN
▪ 20% water

#### 2.2.9 Mobile phase A2

▪ 1 µM NH4F in water

#### 2.2.10 Mobile phase B2

▪ 1 µM NH4F
▪ 65% MeOH
▪ 30% ACN
▪ 5% water

#### 2.2.11 Digestion buffer

▪ 8 M urea
▪ 50 mM ammonium bicarbonate
▪ pH 8.0

#### 2.2.12 Cleavage cocktail

▪ 95% TFA
▪ 2.5% triisopropylsilane
▪ 2.5% H_2_O

### 2.3 Equipment

• HPLC (for LC-MS/MS): Agilent 1100 system (stack with G1376A binary capillary pump, G1477A micro well-plate autosampler, and G1330A ALS thermostat).
• HPLC (for peptide purification): Agilent 1100 HPLC system (stack with G1322A vacuum degasser, G1311A quaternary pump, G1313A autosampler, G1316A column compartment, G1315B diode array detector, and G1364C analytical fraction collector).
• Mass spectrometer: Thermo Scientific, Thermo Scientific Orbitrap Velos
• Probe homogenizer: Fisherbrand, model no. FB120110
• Peptide synthesis machine: Intavis Multipeptide RSi

## 3 Methods

### 3.1 Sample preparation

Wildtype zebrafish (AB strain) were bred and kept in the University of Ottawa Aquatics core facility at 28°C on a 14:10h light/dark cycle. All the fish were fed twice a day. Sexually mature zebrafish were anaesthetized using buffered 4.5% tricaine, then sacrificed by spinal transection. Brain, pituitary and gonads from 42 females and 12 males were dissected and immediately transferred into 1.5 mL centrifuge tubes containing 200 µL of homogenization solution on ice. To homogenize the tissues, a probe homogenizer was utilized at a power output of 10 Watts for 20 seconds. The homogenization solution dissolves the peptide and steroid hormones, while precipitating the proteins to facilitate analysis. After centrifugation, the resulting homogenate was separated into pellet and supernatant fractions, which were then evaporated using a speed-vac.

### 3.2 Pellet digestion

Dried pellets from the tissue homogenates were solubilized in 200 µL digestion buffer. Dithiothreitol was added to a final concentration of 10 mM and incubated at room temperature for 1hr for reduction. Iodoacetamide was added to a final concentration of 20 mM and incubated at room temperature for 40 min in dark for alkylation. In the end, the sample was diluted 5-fold by 50 mM ammonium bicarbonate and digested by 1 µg trypsin/20 µg sample protein overnight at room temperature.

### 3.3 Solid phase extraction

The solid phase extraction (SPE) plates were prepared for both peptide-steroid co-extraction and protein hydrolysate extraction by packing 10 mg of extraction sorbent into filtered 96-well plates. Prior to extraction, the pre-packed extraction plates were activated by adding 200 µL of activation solution for 5 times, followed by 5 times 200 µL of equilibrium solution. The samples were dissolved and loaded in the equilibrium solution, and the loaded SPE plates were washed another 5 times with 200 µL of the same solution. The elution was performed by adding 20 µL of elution solution for 6 times. The activation elution solutions were flushed through the packed SPE bed by centrifugation at 500 rcf, while the equilibrium solution was flushed at 1000 rcf. The stepwise procedure is presented in **Supplementary File 2**.

### 3.4 LC-MS/MS

For both untargeted proteomics and peptidomics, the system consisted of an Agilent 1100 micro-HPLC system coupled with a Thermofisher LTQ-Orbitrap mass spectrometer equipped with a nano-electrospray interface. Peptide separation was performed on an in-house packed 50 μm × 100 mm analytical column. For proteomics, the gradient was from 5% to 35% mobile phase B in 120 min. The mass spectrometry (MS) method consisted of one full MS scan, followed by data-dependent MS/MS scan of the 5 most intense ions using the LTQ-Orbitrap mass spectrometer. The full MS scan range was 150 to 1500 m/z for peptides and steroids, and 300 to 1700 m/z for digested samples. To improve mass accuracy, all measurements in the Orbitrap mass analyzer were performed with internal recalibration (“Lock Mass”). On the Orbitrap, the charge state rejection function was enabled, with single and “unassigned” charge states rejected for untargeted experiments. The raw files generated by the LTQ-Orbitrap were processed and analyzed using MaxQuant, Version 1.2.2.5 with the Uniprot protein FASTA database, including commonly observed contaminants. The following parameters were used: cysteine carbamidomethylation was selected as fixed modification; methionine oxidation, protein N-terminal acetylation, and enzyme specificity was set to trypsin. Up to two missing cleavages of trypsin were allowed to compensate insufficient enzyme digestion. Precursor ion mass tolerances were 7 ppm, and fragment ion mass tolerance was 0.8 Da for MS/MS spectra. Identified peptide sequences from one protein that were equal to or contained within another protein’s peptide set were grouped together and reported as one protein group. The false discovery rate (FDR) for peptide and protein was set at 1%, and a minimum length of six amino acids was used for peptide identification. Quantification was performed using normalized label-free quantitation (LFQ) intensity.

Untargeted peptidomics followed a similar analysis pipeline. However, we selected methionine oxidation, protein N-terminal acetylation, and no enzyme digestion as modifications, and cysteine carbamidomethylation was not fixed.

For targeted peptide and steroid quantification, the HPLC was run in micro mode with a pre-column splitter on load and elute modes, using an in-house-prepared 50 µm × 150 mm analytical column with 3 µm C18-AQ sorbent from Dr. Maisch. The sample was loaded on the column using 95% mobile phase A (A1 for positive mode, A2 for negative mode) at a flow rate of 6 μL/min for 3 min. Then, we used a gradient of 20-90% B1 for positive mode analysis and a gradient of 50-90% B2 for negative mode analysis. We set the mass spectrometer to run on data-dependent acquisition, with the orbitrap analyzer performing the master scan at resolution 60,000 (FWHM), and the linear ion-trap analyzer performing the fragmentation and secondary MS scan according to the mass list and master scan. The mass list collects all primary ions of analytes of interest. We used Thermo Xcalibur software, version 2.2, for data acquisition and analysis. We confirmed peptide structures by three MS2 ions and steroid structures by one MS2 ion. We plotted chromatograms by accurate MS1 with 10 ppm mass tolerance and 120 sec identification windows, using Genesis peak detection when there were over five data points to form a chromatographic peak. We determined the level by comparing the peak area to the standard curve generated by standard compounds. Steroid standards were commercially available. Peptide standards were prepared by solid phase peptide synthesis and purified by HPLC.

### 3.5 Solid phase peptide synthesis

Standard peptides were synthesized in-house using Fmoc peptide synthesis chemistry. Solid-phase synthesis was carried out on Tentagel resin. For deprotection of the Fmoc group on the N-terminus of each amino acid, 2 times 5 min 20% piperidine treatment was employed. Standard coupling reactions were performed with 4-fold HBTU (0.5 M), 4.4-fold amino acids (0.6 M), and 4-fold NMM (4 M) for 40 mins. Double coupling reactions were used to attach each amino acid for sequences with a length of 15 or below, while triple coupling reactions were used for sequences with a length greater than 15.

At the end of synthesis, the resin was treated with a cleavage cocktail for two hours to harvest crude peptides. To introduce cysteine into the peptide sequences of zebrafish oxytocin and vasopressin, Fmoc-Cys(Acm)-OH was used. The Acm-protected cysteine was oxidized on-resin by suspending the synthesis resin in a 4 M Iodine/DMF solution with mild mixing for 2.5 h, followed by washing with DMF 5 times and with DCM 3 times before cleavage. To introduce pyroglutamic acid (Pyr), Boc-Pyr-OH was used with a standard coupling protocol. The crude peptides were precipitated with ethyl ether on ice, dissolved in 50% ACN water, and lyophilized. Lyophilized crude peptide samples were stored at -20°C for purification.

Peptide purification was performed on an Agilent 1100 HPLC system with Luna Omega 5 µm 100 Å PS C18 RP-HPLC column (250×10 mm) at a flow rate of 4 ml/min. The column oven was set at 50°C. To purify each peptide, the HPLC gradients were adjusted to have the target peptide elute at least 7.5 min after the gradient started. The fraction collector was set to start collection 7.5 min after the gradient started on 400 mAU threshold, 500 mAU/s upslope, and 200 mAU/s downslope according to the 214 nm chromatogram. The collected fractions were chilled in liquid nitrogen for 5 min and lyophilized accordingly. Lyophilized purified peptide powders were stored at -20°C. The molecular weight of each peptide was confirmed on LC-MS.

## 4 Results

### 4.1 Peptide and steroid analysis

A total of 8 peptide and 5 steroid hormones were selected to evaluate the possibility of targeted analysis in unpooled, single tissues harvested from multiple females and males. The supernatants were resuspended and subjected to SPE using in-house prepared cartridges, as described in the methods and **Table 1**. Peptide standards were prepared in-house with Fmoc/tBu chemistry, purified, and confirmed by MS as described above. The level of each analyte was determined by comparing the chromatogram peak areas to standard curves. Chromatograms of 3 representative analytes (vasotocin, also known as fish arginine vasopressin (AVP), gonadotropin releasing hormone 3 (GNRH3) and testosterone) are shown in **Figure 1C**, and chosen for their diversity in size, structure, and hydrophilicity/lipophilicity. The limits of detection and maximum amounts of these and other analytes measured from tissues of both females and males are listed in **Table 2**. This new analysis strategy provides the possibility to measure diverse peptide and steroid hormones in tissue samples as small as a zebrafish pituitary. The performance of the in-house packed SPE cartridge was also tested with the LC-MS/MS system, and the overall recovery rates for the 3 representative analytes were over 85% on 0.01-10 nmol loading.

**Table 1:** Criteria and the optimization ranges for SPE and nano-LC methods development.

	Criteria		Optimization range tested
SPE	Sorbent amount		3, 5, **10**, 20 (mg)
	Elution solution composition		70, **75**, 80, 85 (%ACN)
	Elution volume		20-200 (µL), 20 µL increment (**120**)
Nano-LC	Trap	Trap sorbent size	3, **5** (µm)
		Trap column dimension	50, **75**, 100, 200 µm ID
		Trap flow rate	2-10 (µL/min), 1 µL increment (**6**)
	Analytical	Analytical sorbent size	**3** µm, 5 µm
		Analytical column dimensions	**50**, 75 (µm); **150**, 200 (mm)

The SPE and chromatography methods were optimized using GNRH1, mouse SN, and progesterone as external standards in pooled zebrafish brain and pituitary samples. These compounds were chosen due to their diverse size and hydrophobicity. To account for possible matrix effects, pooled zebrafish brain and pituitary samples were spiked with these external standards for the optimization process. The LC-MS/MS analysis exhibited an intra-day coefficient of variation ranging between 5% and 15% over five consecutive days using 3 injections per day. The complexity of the co-extraction samples posed a challenge because of the potential to damage the chromatography columns. To mitigate this, the final combination of method parameters was optimized, and the final criteria are presented above (bold text). This resulted in the generation of repeatable chromatograms with minimal variation in retention time (within a 5-second range) and peak area (<15%) over 100 injections. In the optimized setup, each 96-well plate contained 84 biological samples and a 12-point standard curve. The standard curve samples were injected both before and after the 84 sample injections, and the trap column was replaced thereafter. The standard curves served as both quantification and quality control standards.

**Table 2:** The maximum levels measured for each compound in unpooled tissues from groups of female (N=42) and male (N=12) zebrafish. The lower limit of detection represents the lowest concentration point of the standard curve.

		Max amount measured in females (pmol/tissue)	Max amount measured in females (pmol/mg tissue)	Max amount measured in males pmol/tissue)	Max amount measured in males (pmol/mg tissue)	Lower limit of detection (pmol/mg tissue)
AVP	Brain	328	58.2	289	40.4	0.5
	Pituitary	4013.5	N/A	1348.5	N/A	
	Gonads	888.5	9.5	305	63.4	
E1	Brain	7936	1407.1	1576.5	220.5	0.5
	Pituitary	1102	N/A	587	N/A	
	Gonads	3487	37.3	922.5	191.8	
E2	Brain	2634	467.0	330	46.2	0.2
	Pituitary	973.5	N/A	587	N/A	
	Gonads	7936	84.9	205.5	42.7	
E3	Brain	4786	848.6	4517.5	631.8	1
	Pituitary	4743	N/A	4785	N/A	
	Gonads	4775.5	51.1	1717	357.0	
GNRH2	Brain	576.5	102.2	1033.5	144.6	0.015
	Pituitary	39.5	N/A	46	N/A	
	Gonads	99.5	1.1	119	24.7	
GNRH3	Brain	1757	311.5	436.5	61.1	0.2
	Pituitary	78.5	N/A	65	N/A	
	Gonads	556.5	6.0	135	28.1	
KISS1	Brain	99.5	17.6	243	34.0	0.03
	Pituitary	53	N/A	32.5	N/A	
	Gonads	141	1.5	86	17.9	
KISS2	Brain	119.5	21.2	117.5	16.4	0.015
	Pituitary	100	N/A	87	N/A	
	Gonads	1516	16.2	182	37.8	
OXT	Brain	5723	1014.7	2432	340.1	1.9
	Pituitary	165997	N/A	65132	N/A	
	Gonads	180.5	1.9	384	79.8	
SNa1-34	Brain	448290	79484.0	180954	25308.3	391
	Pituitary	178126	N/A	113238	N/A	
	Gonads	202403	2164.7	154602	32141.8	
SNb1-31	Brain	204334	36229.4	113950	15937.1	195
	Pituitary	184475	N/A	56069	N/A	
	Gonads	185663	1985.7	53584	11140.1	
11-KT	Brain	44.5	7.9	60	8.4	0.5
	Pituitary	19	N/A	60	N/A	
	Gonads	225.5	2.4	446.5	92.8	

Note that amounts are reported as pmol/mg wet weight for brain and gonads. The pituitary is too small to weigh, so we have presented only pmol/tissue (N/A; not applicable).

### 4.2 Untargeted peptidomics

To perform untargeted peptidomics, we applied the peptide-steroid extracts from pituitary to LC-MS/MS analysis using a shallow gradient and data dependent MS acquisition. We used the acquired MS/MS spectra to search against the zebrafish International Protein Index protein sequence database (Version 3.85) with Maxquant and the LFQ option. Our analysis successfully identified 260 different peptides derived from 168 different proteins (**Supplementary File 1**, **Figure 1D**).

### 4.3 Untargeted proteomics

The protein pellets were subjected to reduction, alkylation, and digestion, and the extracts were further processed using the same SPE procedure as described in the Supplementary. The LC-MS/MS analysis and data processing followed a similar protocol, with trypsinization and alkylation being used as the default methods. Analysis of the resulting data revealed the identification of 1758 proteins, of which 93 were found to be shared with the peptidomics results (**Supplementary File 1**, **Figure 1D**).

### 4.4 Gene set enrichment analysis

We conducted gene set enrichment analysis (GSEA) using precursor proteins and associated peptides from both proteomics and the peptidomics results (**Supplementary File 1**). This analysis led to the identification of 393 pathways from the pituitary protein lysate (P<0.05) and 202 pathways from the peptide (P<0.05) datasets (**Supplementary File 1**). This approach allowed us to investigate biological processes and molecular pathways more comprehensively, providing valuable information for further exploration and interpretation of our results. Interestingly, 123 pathways were discovered in both peptidomics and proteomics experiments, suggesting their importance in the same pituitary tissue. The details of all pathways are in **Supplementary File 1**, and specific examples of hormonal pathways enriched in both datasets are presented in **Table 3**. Given the importance of the pituitary hormones in homeostasis and physiological regulation, detection of some of these were expected.

**Table 3:** Examples of gene sets enriched in the proteomics dataset that are also enriched in the peptidome dataset.

Name	# of Entities	Expanded # of Entities	Percent Overlap	p-value	Jaccard similarity	Hit type
ADCYAP1 Expression Targets	59	133	21	2.04E-04	0.012	Biomarkers
Serotonin/Gq Expression Targets	41	89	23	2.51E-04	0.009	Biomarkers
NPY1R -> CRH/POMC Production	38	161	13	2.58E-04	0.009	Biological Process
GALR1/2/3 -> POMC/NPY Production	33	139	12	6.97E-04	0.008	Signal Processing
POMC Secretion in adenohypophysis	31	56	33	2.65E-10	0.009	Biological Process
Angiotensin-Aldosterone System Activation Scheme	43	74	33	4.97E-12	0.011	Disease
VIP Expression Targets	59	111	19	2.23E-03	0.010	Biomarkers
AVP/Gs -> STAT Expression Targets	22	51	27	5.39E-04	0.006	Biomarkers
Leptin -> STAT Expression Targets	96	139	20	4.43E-04	0.012	Biomarkers
Leptin -> ELK/SRF Expression Targets	87	142	22	1.70E-05	0.014	Biomarkers
GPCRs -> Expression Targets in Brain	38	122	18	3.51E-03	0.010	Biomarkers
POMC Expression Targets	60	105	24	1.88E-05	0.012	Biomarkers

The name of the signaling pathway is given, as well as the number of entities involved in the pathway, number assessed, percent overlap (or coverage in the proteomics dataset), p-value, Jaccard similarity index, and hit type. Proteins detected in the pituitary are most representative of these neuroendocrine signaling pathways.

**Figure 2:**
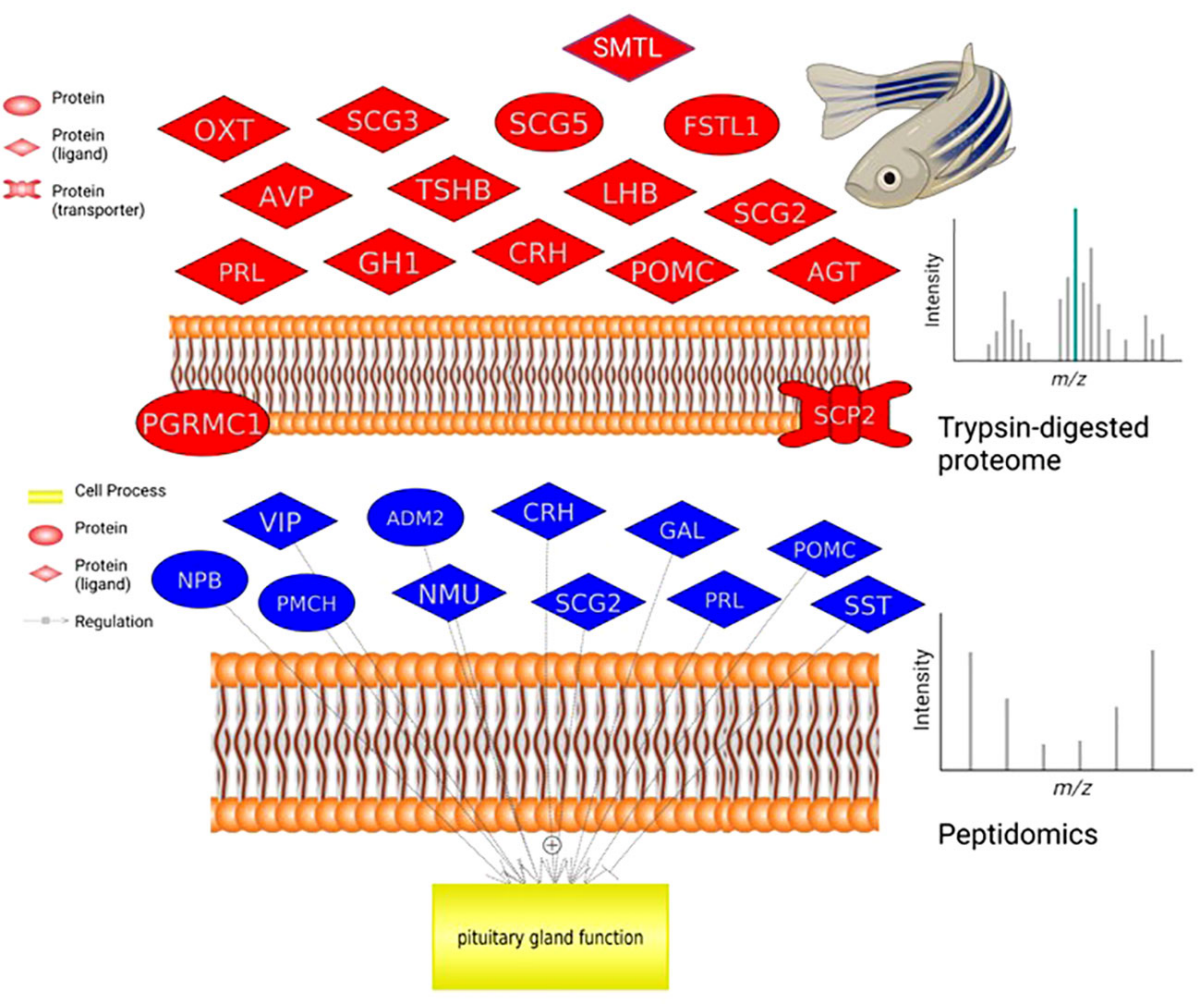
Pituitary proteome and peptidome comparison. Neurohormones identified by proteomics in a single zebrafish that regulate pituitary hormone release (top graph). Neuroendocrine proteins identified by peptidome analysis in a single zebrafish pituitary (bottom graph). For analysis and ease of comparison, we followed nomenclature and abbreviations conventions of HUGO (https://www.genenames.org/): ADM2, adrenomedullin 2; AGT, angiotensinogen; AVP, arginine vasopressin; CRH, corticotropin releasing hormone; FSTL1, follistatin like 1; GAL, galanin and GMAP prepropeptide; GH1, growth hormone 1; LHB, luteinizing hormone subunit beta; NMU, neuromedin U; NPB, neuropeptide B; OXT, oxytocin/neurophysin I prepropeptide; PGRMC1, progesterone receptor membrane component 1; PMCH, pro-melanin concentrating hormone; POMC, proopiomelanocortin; PRL, prolactin; SCG2, secretogranin II; SCG3, secretogranin III; SCG5, secretogranin V; SCP2, sterol carrier protein 2; SST, somatostatin; SMTL, somatolactin; TSHB, thyroid stimulating hormone subunit beta; VIP, vasoactive intestinal peptide.

For example, pathways related to proopiomelanocortin (POMC) production, secretion and action are well represented in the data (**Figure 2**, **Table 3**). A lesser studied aspect of pituitary function is that of leptin action via STAT, ELK/SRF expression targets (**Table 3**). In fish, leptin can regulate prolactin (PRL) (11) and the gonadotropins (12). We also noted that enzymatic processing of classical hormones such as PRL can give rise to angiogenic peptides (13, 14) that were also found in the pituitary gland.

A key example of the utility of the methodology is the detection and quantification of the emerging reproductive hormone secretoneurin (SN). It is derived from prohormone convertase-mediated processing of the larger secretogranin-2 (SCG2) precursor which was detected in the proteomic samples (**Figure 2**). In zebrafish and other teleosts there are 2 precursor forms, SCG2a and SCG2b, respectively giving rise to SNa1-34 and SNb1-31 which we synthesized and then quantified in brain, pituitary and gonads of both females and males (**Table 2**). TALEN-mediated frameshift mutation of the zebrafish SCG2a and SCG2b genes led to reduced levels of sexual activity, suboptimal ovulation and egg laying, fertility, and embryonic survival 24-hr postfertilization (10). The SN peptides stimulate LH production and release (15). Gene set enrichment analysis (GSEA) was conducted in Pathway Studio 12.0 (Elsevier). A total number of 2,095 proteins (proteomics) and 202 proteins (based on peptidomics) were mapped into the program for zebrafish using the official gene name. GeneCards (www.genecards.org/) was used to manually retrieve mammalian homologs for mapping. Gene set enrichment analysis proceeded with 1000 permutations to generate the distributions for statistical testing (permutation test) using a Kolmogorov-Smirnov Distribution test. Parameters were as follows: p-value ≤ 0.05, maximum number of networks shown in result = 1000 with a minimum network overlap of 5 entities. The enrichment identifies cell processes and signaling pathways most represented in the data sets.

### 4.5 Targeted protein analysis

We also analyzed pituitary protein lysates using targeted proteomics to investigate the presence of specific proteins, namely the subunits of luteinizing hormone (LH). Critically, LH is a dimeric pituitary hormone responsible for driving ovulation in all vertebrate animals (16) and inappropriate LH secretion can lead to various fertility problems (7). It is traditionally measured by radiometric or enzyme-liked immunoassays, with associated challenges (3, 17, 18). There is currently no specific immunoassay for zebrafish LH. As an alternative, we optimized proteomic digestion steps and then identified two fragments from the glycoprotein hormones alpha polypeptide (CGA) (CGA 48-62 and CGA 27-38) and two fragments from the luteinizing hormone subunit beta (LHB) (LHB 9-29 and LHB 30-43) for the detection of LH (**Figure 1B**).

## 5 Discussion

Our method enables the analysis of peptides, steroids and proteins at the same time. This lays the foundation for comprehensive assessment of hormonal pathways in a single sample, allowing for direct comparisons and improved data interpretation. The performance of the optimized method for simultaneously measuring both peptides and steroids was similar to previous reports on peptide or steroid analysis individually (19–21). The proposed strategy effectively addresses the problematic issue of separately isolating and detecting small lipophilic versus hydrophilic endogenous hormones.

In this proof-of-principle experiment, we targeted 6 well-known peptide and 5 steroid hormones plus the reproductive peptides SNa1-13 and SNb1-31 in brain, pituitary and gonads. We were able to quantify these 13 hormones in most samples, given the dynamic range of our standard curves. There were sex differences both in the total amounts produced in a tissue, or when adjusted for wet weight. While sex differences in steroids may be somewhat expected based on published literature (e.g., 22, 23), the peptides presented have never been measured in zebrafish tissues to our knowledge. The SNa and OXT peptides were found in high quantities, especially in the pituitary. This supports previous anatomical observations on the colocalization of OXT and SNa in goldfish magnocellular neurons and their projections to the anterior and posterior pituitary (24). In rat, the highest levels of SN were quantified in the median eminence (25), the functional equivalent to direct innervation of the anterior pituitary of teleost (26).

An impressive number of other pituitary peptides were evident from the untargeted peptidomics analysis. Our method was also designed for the comparison of the proteome and peptidome of small tissues, such as the pituitary. We were able to match precursor proteins with some of the peptides generated. As precursor processing by prohormone convertases is physiologically regulated, our method will be useful to study the generation on known hormonal peptides, but also the discovery of novel fragments. Gene set enrichment analyses identified signaling pathways common to both the proteomic and peptidome datasets. The main limiting factor for quantification of novel hormones is the availability of commercial standards, and but in many cases, as we have shown with SN and other teleost peptides, this is easily overcome with high quality synthesis and purification steps. Given that there are so far, ~20 stimulatory and ~10 inhibitory neuropeptides, 3 amines, 3 amino acid neurotransmitters and many steroids implicated in the control of teleost reproduction (16), the method holds promise for comprehensive assessment of natural variations, or in response to gene editing and environmental perturbations.

Peptide and steroid measurements are important not only in biomedical research but also in industries such as food safety evaluation and environmental assessment. For example, in dietary supplements, collagen hydrolysate peptide profile consistency is important for nutrition, while determining steroid levels is important for safety (27, 28). If the peptide profile and steroid levels are tested at the same time, food safety authorities could make inspections more efficient and less costly. Monitoring the levels of steroid hormones, especially synthetic and potent analogs, is crucial to monitoring pollution and endocrine-disrupting chemicals in the aquatic environment (29). Additionally, formylmethionyl-peptides are key indicators of the level of bacteria (30). Measuring these two categories of compounds together would permit ecotoxicologists to better understand chemical threats to ecosystems and human health. All of these will require further development.

The SPE method can be efficiently used to extract peptides and steroid samples for other assay systems. We have shown it to be compatible with a commercial estradiol ELISA kit, being validated using aromatase mutant zebrafish lines (31). Having reusable SPE plates in a small-scale research laboratory that serves multiple different ELISA tests can not only save on research funds but also significantly decrease the plastic waste it generates. In addition, it can decrease the variability between different batches of SPE kits. The extraction and analysis methods employ the 96-well plate platform, thus making it feasible to motorize sample processing procedure and downstream analysis. With minimum optimization, this package of methods can be used for high throughput routine analysis in research institutes or clinical test centers.

## Data Availability

All data are presented in the main article or in the supplemental files. Any other reasonable queries should be directed at the corresponding authors.
