# Corrigendum: Simultaneous extraction and detection of peptides, steroids, and proteins in small tissue samples

**DOI:** 10.3389/fendo.2024.1476509

**Published:** 2024-09-05

**Authors:** Chunyu Lu, Di Peng, W. C. K. Udeesha Erandani, Kimberly Mitchell, Christopher J. Martyniuk, Vance L. Trudeau

**Affiliations:** ^1^ Department of Biology, University of Ottawa, Ottawa, ON, Canada; ^2^ Department of Physiological Sciences, University of Florida, Gainesville, FL, United States

**Keywords:** protein measurement, peptide quantification, steroid detection, LCMS (liquid chromatography-mass spectrometry), solid phase extraction

In the published article, there was an error in the author list, and authors Chunyu Lu and Di Peng were not identified as co-first authors. A footnote to the author list has been included in the article:

“These authors share first authorship.”

The authors apologize for this error and state that this does not change the scientific conclusions of the article in any way. The original article has been updated.

