# Supplementary material for: Simultaneous extraction and detection of peptides, steroids, and proteins in small tissue samples
Source: Front Endocrinol (Lausanne). 2023 Oct 9;14:1266985. doi: 10.3389/fendo.2023.1266985 (PMC10593444; doi:10.3389/fendo.2023.1266985)
Supplement: Supplementary file 2 [file Table2.docx]

Supplemental File 2

Simultaneous extraction and detection of peptides, steroids, and proteins in small tissue samples

Chunyu Lu^1^, Di Peng^1^, W.C.K. Udeesha Erandani^1^, Kimberly Mitchell^1^, Christopher J. Martyniuk^2^ & Vance L. Trudeau^1*^

^1^Department of Biology, University of Ottawa, Ottawa, ON K1N 6N5, Canada

^2^Department of Physiological Sciences, University of Florida, Gainesville, FL 32611

**Stepwise Procedure for Solid Phase Extraction.**

***Packing an SPE plate***

1. Measure 1 g of sorbent, and place in 20 ml flat bottom glass flask with a magnetic stir bar in.
2. Add 10 ml of HPLC grade acetonitrile and agitate with magnetic mixing to generate an evenly distributed slurry.
3. Place a filtered well plate on the waste collection tray.
4. Aliquot 100 µL of the slurry in each well of the filtered well plate by 100 µL or 200 µL pipettor with the flask agitation on.

The remaining slurry can be stored in the flask with airtight cap at room temperature. The stir bar can remain in the flask with to agitation. When packing another SPE plate, sorbent and acetonitrile can be added to the flask by the same ratio. It is not necessary to empty or clean the flask in between two experiments. The SPE plate can be packed on an independent day to the actual extraction experiment. The plate can be stored at room temperature with an aluminum foil cover to prevent dust.

***Extraction***

Pre-condition swing bucket centrifuge to 4 ºC.

Prepare equilibrium buffer (0.2% formic acid, 5% acetonitrile, 95% water); activation buffer (0.2% formic acid, 50% acetonitrile, 50% water); and elution buffer (0.2% formic, 75% acetonitrile, 25% water). If the plate is to be regenerated and reused, prepare regeneration buffer (0.2% formic acid, 95% MeOH, 5% water). Resuspend each lyophilized sample in a 200 µL equilibrium buffer. Keep samples on ice and proceed with the protocol below.

1. Place the SPE plate on top of the waste tray.
2. Add 200 µL activation buffer to each SPE well.
3. Centrifuge on 500 rcf for 1 min.
4. Empty waste tray
5. Proceed with steps 1-4 for 4 times.
6. Place the SPE plate on top of the waste tray.
7. Add 200 µL equilibrium buffer to each SPE well.
8. Centrifuge on 1000 rcf for 1 min.
9. Empty waste tray
10. Proceed with steps 6-9 for 4 times.
11. Place the SPE plate on top of the waste tray.
12. Load each sample in each well of the SPE plate.
13. Centrifuge on 1000 rcf for 1 min.
14. Empty waste tray
15. Proceed with steps 6-9 for 5 times.
16. Place SPE plate on top of HPLC sampler plate.
17. Add 20 µL elution buffer to each SPE well.
18. Centrifuge on 500 rcf for 1 min.
19. Proceed with steps 17-18 for 5 times with the same collection plate.
20. Evaporate the solvent from the sampler plate with a swing bucket centrifuge connected to lyophilizer.
21. Seal lyophilized plate and store at -20 ºC for LCMS analysis
22. Place the SPE plate on top of the waste tray.
23. Add 200 µL regeneration buffer to each SPE well.
24. Centrifuge on 500 rcf for 1 min.
25. Empty waste tray
26. Proceed with steps 21-24 fo 4 times.
